# A Unique Gene-Silencing Approach, Using an Intelligent RNA Expression Device (iRed), Results in Minimal Immune Stimulation When Given by Local Intrapleural Injection in Malignant Pleural Mesothelioma

**DOI:** 10.3390/molecules25071725

**Published:** 2020-04-09

**Authors:** Hidenori Ando, Noriko Saito-Tarashima, Amr S. Abu Lila, Nozomi Kinjo, Taro Shimizu, Yu Ishima, Noriaki Minakawa, Tatsuhiro Ishida

**Affiliations:** 1 Department of Pharmacokinetics and Biopharmaceutics, Institute of Biomedical Sciences, Tokushima University, 1-78-1, Sho-machi, Tokushima 770-8505, Japan; h.ando@tokushima-u.ac.jp (H.A.); c401503025@tokushima-u.ac.jp (N.K.); shimizu.tarou@tokushima-u.ac.jp (T.S.); ishima.yuu@tokushima-u.ac.jp (Y.I.); 2 Department of Bioorganic Chemistry, Institute of Biomedical Sciences, Tokushima University, 1-78-1, Sho-machi, Tokushima 770-8505, Japan; noriko.tarashima@tokushima-u.ac.jp (N.S.-T.); minakawa@tokushima-u.ac.jp (N.M.); 3 Department of Pharmaceutics and Industrial Pharmacy, Faculty of Pharmacy, Zagazig University, Zagazig 44519, Egypt; amr_selim78@yahoo.com; 4 Department of Pharmaceutics, College of Pharmacy, Hail University, Hail 81442, Saudi Arabia

**Keywords:** cationic liposomes, innate immunity, intelligent RNA expression device (iRed), RNA interference, shRNA

## Abstract

Background: We have recently introduced an intelligent RNA expression device (iRed), comprising the minimum essential components needed to transcribe short hairpin RNA (shRNA) in cells. Use of iRed efficiently produced shRNA molecules after transfection into cells and alleviated the innate immune stimulation following intravenous injection. Methods: To study the usefulness of iRed for local injection, the engineered iRed encoding luciferase shRNA (Luc iRed), complexed with cationic liposomes (Luc iRed/liposome-complexes), was intrapleurally injected into an orthotopic mesothelioma mouse model. Results: Luc iRed/liposome-complexes markedly suppressed the expression of a luciferase marker gene in pleurally disseminated mesothelioma cells. The suppressive efficiency was correlated with the expression level of shRNA within the mesothelioma cells. In addition, intrapleural injection of iRed/liposome-complexes did not induce IL-6 production in the pleural space and consequently in the blood compartment, although plasmid DNA (pDNA) or dsDNA (the natural construct for iRed) in the formulation did. Conclusion: Local delivery of iRed could augment the in vivo gene silencing effect without eliciting pronounced innate immune stimulation. Our results might hold promise for widespread utilization of iRed as an RNAi-based therapeutic for intracelial malignant cancers.

## 1. Introduction

RNA interference (RNAi), a biological process in which RNA molecules inhibit gene expression or translation, has emerged as a powerful tool for studying gene function and holds promise for the development of new gene silencing therapies [1,2]. Two key RNAi molecules, small interfering RNA (siRNA) and short hairpin RNA (shRNA), produced from plasmid DNA (pDNA), have gained considerable interest as tools for gene silencing [3]. Nevertheless, each of them has shortcomings. In the absence of chemical modifications, siRNA has lower chemical stability than pDNA, and can trigger off-target effects [4,5]. As well, shRNA, produced from pDNA, has lower expression efficiency. In addition, pDNA induces innate immune responses when it contains CpG motifs in its sequence [6]. Accordingly, alternative transfection techniques are still needed.

Several approaches have been adopted to increase the stability of RNAi molecules, to maximize their in vivo activities, and to attenuate host immune stimulation and/or off-target effects. For instance, chemical modifications that include replacement of uridines with 2’-fluoro uridines or 2’-O-methyl uridines can effectively prevent innate immune responses against siRNA and reduce off-target silencing effects [7]. In addition, incorporation of two nucleotide overhangs at the 3’ end of only the antisense strand in siRNA can reduce off-target sequences [8,9]. However, it has proven quite difficult to modify the pDNA structure to attenuate the innate immune responses, although the chemical stability of pDNA is higher than that of siRNA/shRNA. Minimal-sizing, or the modification of nucleotide units of pDNA, can affect the transcription step that results in the production of target shRNA after being incorporated into nuclei.

As a novel approach to alleviate the innate immune stimulation of pDNA molecules, we have developed a new artificial DNA construct, i.e., “intelligent RNA expression device (iRed)”, and studied its ability to induce RNAi gene-silencing [10]. iRed contains the minimum number of components needed for shRNA production in cells, including a U6 promoter and an shRNA-encoding region, in which cognate 4’-thio derivatives of thymine (T), guanine (G), cytosine (C) or adenine (A) nucleotide units were substituted for natural nucleotides [11,12]. The engineered iRed produced the expected shRNA when transfected into cells, followed by induction of a specific RNAi-based gene silencing effect [10]. In addition, after intravenous injection, the iRed complexed with cationic liposomes (iRed/liposome-complexes) modified with polyethylene glycol (PEG) efficiently alleviated innate immune stimulation owing to both the downsizing of the minimal structure required to transcribe shRNA and the presence of the 4’-thiomodification [10].

Recently, we have emphasized the potential of intracelial injection of RNAi molecules for the treatment of malignant disseminated cancers developed in either the pleural cavity or the peritoneal cavity. In an orthotopic pleural mesothelioma murine model, survival times were increased by intrapleural injections of shRNA/liposome-complexes against thymidylate synthase (TS), which efficiently sensitized tumor cells to the cytotoxic effect of the anticancer drug, pemetrexed, by down-regulating the expression of TS mRNA in the disseminated tumor cells [13]. In addition, intraperitoneal injection of TS shRNA/liposome-complexes markedly prolonged the survival time in a peritoneally disseminated gastric cancer murine model [14]. However, the injected shRNA still holds the potential to activate the innate immune system [15,16,17]. Hence, the intracelial injection of RNAi molecules including iRed, engineered to generate shRNA in cells, could be an effective approach for the treatment of malignant cancer developed in the coelomic cavity while simultaneously attenuating RNAi-associated innate immune stimulation. In this study, to expand the usefulness of iRed, we studied the effectiveness of engineered dSC iRed, in which cytosine (C) nucleotide unit was substituted by cognate 4′-thio derivatives, encoding luciferase shRNA (Luc iRed) given by local intracelial injection in a luciferase-expressing orthotopic murine model of pleural mesothelioma. The luciferase activity in the disseminated tumors was determined, and IL-6 in both serum and the pleural cavity was assessed as an indicator for possible innate immune stimulation. 

## 2. Results

### 2.1. Preparation of DNAs/Liposome-Complexes

**Figure 1:**
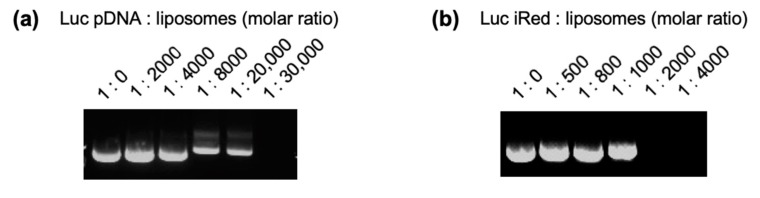
Electrophoretic profile of DNA/liposome-complexes. (**a**) Electrophoretic profile of Luc pDNA/liposome-complexes at different ratios ranging from 1:2000 to 1:30,000. (**b**) Electrophoretic profile of Luc iRed/liposome-complexes at different ratios ranging from 1:500 to 1:4000. The absence of DNA bands indicates that almost 100% of the DNA was associated with cationic liposomes. The lipid composition of cationic liposomes was DOPE, cholesterol and DOTAP (3:3:4 as molar ratio).

To evaluate the in vivo gene silencing effect of DNAs; including pDNA, dsDNA and iRed, cationic liposomes were used as delivery vehicles to increase the stability of loaded DNAs and to enhance internalization into cells [18]. The construct images of DNAs are shown in Supplementary Figure S1. To confirm the binding efficiency of DNA to cationic liposomes, the presence of free unbound DNA was evaluated by electrophoresis analysis after DNA was mixed with cationic liposomes. As shown in Figure 1a, free Luc pDNA bands were clearly observed at pDNA:cationic liposomes molar ratios up to 1:20,000, but they disappeared at a molar ratio of 1:30,000, indicating that Luc pDNA was completely complexed with cationic liposomes at a molar ratio of 1:30,000. In contrast, (Figure 1b), free Luc iRed bands were only observed at iRed:cationic liposomes molar ratios lower than 1:2000, indicating that Luc iRed was completely bound when the molar ratio was much lower than that for Luc pDNA. The difference on the molar ratio for complexation might be due to the difference in the molecular weights of Luc pDNA and Luc iRed. Luc iRed comprises of only the U6 promoter region and the Luc shRNA-expressing region of Luc pDNA [10], with a molecular weight of ca. 230 kDa (ca. 370 bp). On the other hand, Luc pDNA has a molecular weight of ca. 4200 kDa (ca. 6820 bp). In terms of the ratio of the positively charged amine groups of cationic liposomes to the negatively charged phosphate groups of DNAs (N/P ratio), Luc iRed/liposome-complexes at a molar ratio of 1:2000 have an N/P ratio of 1.08, and Luc pDNA/liposome-complexes at a molar ratio of 1:30,000 have an N/P ratio of 0.88. This indicates that when N/P ratios of cationic liposomes to DNAs are approximately 1, the DNAs/liposome-complexes including all DNA molecules are adequately prepared. The physicochemical characteristics of the DNAs/liposome-complexes are listed in Table 1. The particle size of the prepared DNAs/liposome-complexes was larger than that of cationic liposomes, and the zeta potentials were lower than that of cationic liposomes. 

### 2.2. In Vitro Gene Silencing Effect of shRNA-Expressing DNAs 

**Figure 2:**
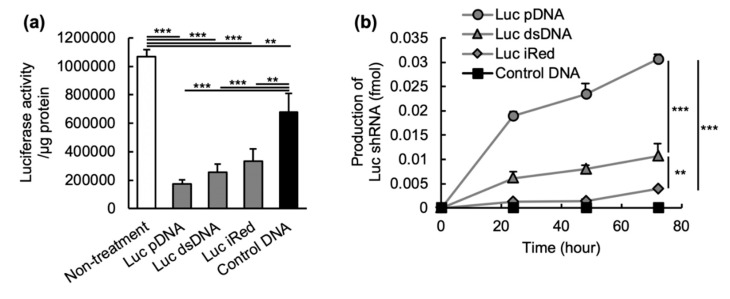
In vitro luciferase gene silencing activities of different DNAs. (**a**) RNAi activity of Luc pDNA, Luc dsDNA, Luc iRed and control DNA. MSTO-211H-Luc cells were transfected with each DNAs using Lipofectamine^®^ 2000 at 0.5 pmol/well according to the following procedure: 2.1 µg Luc pDNA, 0.115 µg Luc dsDNA or 0.115 µg Luc iRed was mixed with 10 µL Lipofectamine^®^ 2000 reagent and then transfected into the cells. Data are reported as mean ± SD (*n* = 3). Significant differences are represented as ** *p* < 0.01, *** *p* < 0.001. (**b**) The amount of shRNA molecules transcribed from different DNAs. The shRNAs were extracted from the cells post-transfection with DNAs using a Lipofectamine^®^ 2000 reagent (0.5 pmol/well) at the indicated time points. The procedure to transfect the DNAs into cells was the same as Figure 2a. Data are reported as mean ± SD (*n* = 3). Significant differences are represented as ** *p* < 0.01, *** *p* < 0.001.

iRed was originally designed to produce shRNA following transfection into cells and, consequently, suppress target mRNA expression. The gene silencing activity of iRed, generating shRNA against luciferase (Luc), was compared with Luc pDNA and Luc dsDNA in human pleural mesothelioma cells stably expressing firefly luciferase (MSTO-211H-Luc). To transfect DNAs into cells, Lipofectamine^®^ 2000, a transfection reagent, was used to clarify the in vitro gene-silencing potential of each DNAs. Luc iRed, Luc pDNA and Luc dsDNA significantly decreased the luciferase activity in the cells compared to control DNA, a dsDNA encoding non-specific shRNA; the rank order of RNAi activity was Luc pDNA > Luc dsDNA > Luc iRed (Figure 2a). To gain more insight into the gene silencing effect of DNAs, the amount of Luc shRNA transcribed from DNAs was quantified (Figure 2b). Control, non-specific DNA did not result in the expression of any target shRNA for luciferase (Luc shRNA). Luc pDNA, Luc dsDNA and Luc iRed all resulted in the expression of Luc shRNA in a dose-dependent manner for up to 72 h. The rank order of production of Luc shRNA was Luc pDNA >> Luc dsDNA > Luc iRed. The amount of expressed Luc shRNA was correlated with the in vitro suppression of luciferase activity as shown in Figure 2a. 

### 2.3. In Vivo Gene Silencing Effect of shRNA-Expressing DNAs Formulated in DNA/liposome-Complexes

**Figure 3:**
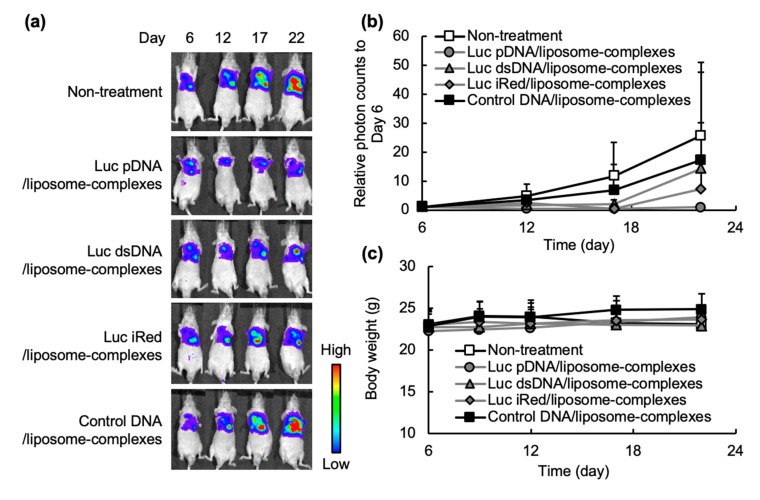
In vivo evaluation of RNAi activities of various different DNAs formulated in DNA/liposome-complexes. Orthotopic mesothelioma-bearing mice were intrapleurally injected with two doses of Luc pDNA/liposome-complexes, Luc dsDNA/liposome-complexes, Luc iRed/liposome-complexes or control DNA/liposome-complexes (10 µg DNA/mouse/day) at day 7 and at day 10 after inoculation of tumor cells. At days 6, 12, 17 and 22 after cell inoculation, the luciferase activity of the pleural tumor was visualized using an in vivo imaging system (IVIS). (**a**) Time course of bioluminescence intensities. (**b**) Time course of luciferase activity expressed as relative photon counts compared to that at day 6 post tumor cell inoculation. (**c**) The body weight of the mice determined from day 6 to day 22 following tumor cell inoculations. In the (**b**,**c**), data are reported as the mean ± SD (*n* = 5).

In order to evaluate the in vivo gene silencing effect of different DNAs formulated in DNA/liposome-complexes, murine models of luciferase-expressing orthotopic mesothelioma (MSTO-211H-Luc cells) were intrapleurally injected with Luc pDNA/liposome-complexes, Luc dsDNA/liposome-complexes, Luc iRed/liposome-complexes or control DNA/liposome-complexes (10 µg DNA/mouse/day) on day 7 and day 10 post-inoculation of tumor cells. At selected time points post-inoculation, the luciferase bioluminescence for the inoculated tumor cells was monitored using an in vivo imaging system (IVIS; Figure 3a) and photon counts were quantified (Figure 3b). The bioluminescence in the pleural cavities of untreated control mice increased with time, indicating the aggressive growth of the inoculated cells. In the mice treated with control DNA/liposome-complexes, similar luciferase activities to those of untreated mice were observed. In the mice treated with Luc pDNA/liposome-complexes, the lowest luciferase activities were observed. The rank order of gene silencing activity was Luc pDNA > Luc iRed > Luc dsDNA. In order to compare the gene-silencing effect of each DNA/liposome-complexes corrected with molar amount, the inhibition rate of DNAs/liposome-complexes was calculated from the data in Figure 3b, which is corrected with injected molar amounts of DNAs/liposome-complexes; 2.4 pmol/mouse Luc pDNA/liposome-complexes, 43.5 pmol/mouse Luc dsDNA/liposomes-complexes and 43.5 pmol/mouse Luc iRed/liposome-complexes, and the data was shown in Table 2. The inhibition rate of Luc pDNA/liposome-complexes was significantly higher than that of Luc dsDNA/liposomes-complexes and Luc iRed/liposome-complexes. In addition, no substantial body weight loss was observed in all mice during the treatment period (Figure 3c), suggesting that the dose and treatment schedule were tolerable.

### 2.4. Induction of Innate Immune Reactions Following Intrapleural Injections of shRNA-Expressing DNAs Formulated in DNA/Liposome-Complexes

**Figure 4:**
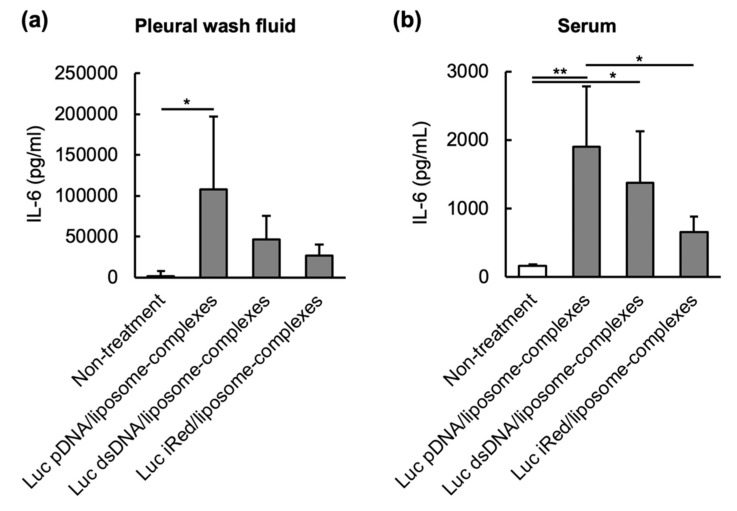
IL-6 production induced by different DNAs, formulated in DNA/liposome-complexes, injected intrapleurally. Orthotopic mesothelioma-bearing mice were intrapleurally injected with Luc pDNA/liposome-complexes, Luc dsDNA/liposome-complexes or Luc iRed/liposome-complexes (10 µg DNA/mouse) on day 7 after tumor cell inoculation. At 4 h post injection, IL-6 production level was measured in (**a**) pleural wash fluid and (**b**) serum. Data are reported as the mean ± SD (*n* = 5). Significant differences are represented as * *p* < 0.05, ** *p* < 0.01.

Undesired immune stimulation by nucleic acid is one of obstacles to develop nucleic acid-related therapeutics. In this study, the immunostimulatory effects of DNAs/liposome-complexes following intrapleural local injection was assessed. IL-6 levels, as markers of innate immune stimulation, were determined in both pleural wash fluid (Figure 4a) and serum (Figure 4b) in mice intrapleurally treated with Luc pDNA/liposome-complexes, Luc dsDNA/liposome-complexes or Luc iRed/liposome-complexes. Intrapleural injection of Luc pDNA/liposome-complexes caused significant IL-6 production in the pleural wash fluid, while both Luc dsDNA/liposome-complexes and Luc iRed/liposome-complexes slightly, but not significantly, increased IL-6 level compared to non-treatment group due to their retention in the cavity. The serum IL-6 level, was correlated to IL-6 production in the pleural cavity, and was increased in all mice treated with Luc pDNA/liposome-complexes, Luc dsDNA/liposome-complexes and Luc iRed/liposome-complexes. The rank order of serum IL-6 levels was Luc pDNA/liposome-complexes > Luc dsDNA/liposome-complexes > Luc iRed/liposome-complexes. These results indicate that iRed, having down-sizing and 4’-thiomodification of the original pDNA, had the weakest immune stimulative activity among three DNAs (pDNA, dsDNA and iRed).

## 3. Discussion

RNAi has emerged as a powerful tool for studying gene function and holds promise for the development of gene therapies [19,20]. We developed a unique DNA construct, intelligent RNA expression device (iRed), which enables the expression of a targeted shRNA in cells after transfection [10]. iRed comprised the minimal structure required to transcribe shRNA with 4’-thiomodification, which synergistically alleviated innate immune responses following its intravenous injection [10]. Our previous report has clarified the potency of the iRed on producing a gene-silencing effect using malignant pleural mesothelioma mouse model by transfection with an in vivo transfection reagent (TurboFect^TM^ Transfection Reagent, Thermo Fisher Scientific), which is impossible to move into clinical application in the future. Therefore, in the present study, we prepared iRed/liposome-complexes and evaluated the applicability/efficacy of iRed following local administration in a pleurally disseminated orthotopic mesothelioma mouse model. The formulations of liposome and lipid nanoparticle are widely used in clinical settings as a drug delivery carrier, e.g., Doxil^®^, Ambisome^®^ and Onpattro^®^. Our present study showed that Luc iRed formulated in DNA/liposome-complexes successfully induced an efficient gene silencing effect against pleurally disseminated tumors (Figure 3) as a consequence of the expression of therapeutic levels of specific Luc shRNA within the cells (Figure 2b). In addition, Luc iRed/liposome-complexes alleviated the innate immune stimulatory effect of Luc pDNA/liposome-complexes following intrapleural injection (Figure 4a,b). We have already reported that local intrapleural administration of RNAi therapeutics (i.e., shRNA against TS) could efficiently suppress malignant pleural mesothelioma tumor progression [13]. Taken together, intrapleural treatment with iRed, formulated in DNA/liposome-complexes, might be a promising therapeutic approach for the treatment of malignant pleural mesothelioma via silencing a target gene while simultaneously attenuating RNAi-associated innate immune stimulation.

Efficient delivery of RNAi molecules into cells is a crucial determinant for the effective regulation of target genes [21,22]. Viral vectors and non-viral nanoparticulate systems are most commonly used vehicles for the delivery of RNAi molecules [23,24,25]. Viral vectors represent the most efficient gene delivery vehicles; however, their immunogenicity and toxicity limit their clinical use [26]. In this study, non-viral cationic liposomes were used to formulate DNA/liposome-complexes. An in vivo gene silencing experiment in an orthotopic tumor model showed that cationic liposomes efficiently delivered the Luc iRed into the tumor cells as manifested by the substantial decrease in luciferase activity of tumor cells inoculated into the pleural cavity (Figure 3a,b). Cationic lipids when used as liposomal delivery systems are hypothesized to undergo a phase transition from a lamellar phase to hexagonal packing in the acidic environment of cellular endosomes, promoting endosomal release of RNAi from the delivery vehicles [27]. This might account for the enhanced iRed-caused RNAi in vivo and thereby efficient gene silencing activity (Figure 3a,b).

In order to gain further insight into the potential of iRed to induce RNAi, the in vitro gene silencing activity and shRNA expression induced by iRed were assessed. Under equimolar condition, all DNAs including Luc pDNA, Luc dsDNA and Luc iRed showed suppression of luciferase activity in MSTO-211H-Luc cells (Figure 2a) and their activity was closely correlated with the expression level of the corresponding Luc shRNA (Figure 2b). The rank order of in vitro shRNA expression induced by these DNAs was also highly correlated to their in vivo gene-silencing (Figure 3b). iRed showed weakest in vitro gene silencing effect among three DNAs (Figure 2a), but it still showed in vivo sufficient gene silencing to have a therapeutic effect (Figure 3a,b).

We demonstrated that the shRNA formulated in shRNA/liposome-complexes prevents shRNA degradation in pleural fluid, and the shRNA/liposome-complexes remain in the pleural cavity for an extended period of time following intrapleural injection [28]. A long retention would increase the opportunity for DNAs/liposome-complexes to interact with the targeted cells and transfect DNAs into the cells. In addition, after the transfection into cells, RNAi molecules enzymatically suppress the expression of target mRNA by a specific RNA-degradative enzyme, Argonaute 2 [29]. The amount of Argonaute 2 would be a rate-limiting factor to determine gene-silencing by RNAi molecules. Therefore, in this study, although the production level of shRNA from iRed was weaker than the other DNAs, the gene-silencing activity must have been high enough to suppress the expression of target mRNAs. Taken together, our results showed that iRed has a potential to exert a therapeutically relevant gene-silencing effect via production of target shRNA following cell uptake.

Besides their potent gene silencing effect, many reports have demonstrated that non-chemically modified nucleic acid molecules activate the innate immune system [30,31,32], which is an undesirable side effect during clinical use. Innate immune responses are characterized by an induction of cytokines, small signaling molecules such as interferons (IFNs), interleukins (ILs) and tumor necrosis factor-α (TNF-α), which subsequently induce tissue inflammation, allergic responses and tissue damage [33,34]. In the present study, Luc pDNA/liposome-complexes and, to a lesser extent, Luc dsDNA/liposome-complexes triggered the production of IL-6 in pleural cavity (Figure 4a). IL-6 was then transferred from the pleural cavity to the blood compartment (Figure 4b), which might cause adverse effects, as described above. In contrast, Luc iRed/liposome-complexes induced a lower level of IL-6 in both the pleural cavity (Figure 4a) and in serum (Figure 4b). These results confirm that iRed is a safer nucleic acid construct than pDNA and dsDNA, which have larger molecular weights.

In this study, we demonstrated that the iRed system induced a therapeutically relevant gene-silencing effect, after producing targeted shRNA following intrapleural injection into an orthotopic pleural mesothelioma mouse model, with minimal innate immune stimulation. Our present study indicates that local injection of iRed formulated in DNA/liposome-complexes could be an advantageous approach to treat malignant tumors in body cavities, including the pleural cavity and the peritoneal cavity, without precipitation adverse effects related to nonspecific immune stimulation.

## 4. Materials and Methods 

### 4.1. Materials

Natural dNTPs were purchased from GE Healthcare Japan (Tokyo, Japan). 2’-Deoxy-4’-thionucleoside triphosphates (dSNTPs) were prepared as previously described [11,18]. Oligonucleotides were obtained from FASMAC (Kanagawa, Japan). Dioleoyl phosphatidylethanol- amine (DOPE) and 1,2-dioleoyl-3-trimethylammonium-propane (DOTAP) were obtained from NOF Corporation (Tokyo, Japan). Cholesterol and D-luciferin potassium salt were purchased from Wako Pure Chemical (Osaka, Japan). All other reagents were of analytical grade.

### 4.2. Construction of shRNA-Expression pDNA

Oligonucleotides having shRNA and terminator coding sequences were ligated into the BamHI and EcoRI sites of an shRNA expression cassette containing the U6 promoter (RNAi-Ready pSIREN-RetroQ, Clontech, CA, USA) according to the manufacturer’s instruction. The inserted sequences are summarized in Supplementary Table S1.

### 4.3. Preparation of Intelligent shRNA-Expression Device (iRed) and shRNA-Expressing Natural Device (Double Strand DNA; dsDNA)

iRed was prepared using the shRNA-expression pDNA as a template for PCR as previously described [10]. The U6 promoter and shRNA sequences in pDNA were amplified in 20 μL of the KOD buffer containing KOD Dash DNA polymerase (0.05 unit/μL, TOYOBO, Osaka, Japan), pDNA template (0.1 fmol/μL), 200 μmol/L dNTPs and 0.5 μmol/L of primers. The reaction mixture was gently vortexed, and the DNA was PCR amplified using a thermal cycler as previously reported [10]. For the preparation of iRed, the PCR amplification was performed in the reaction mixture containing dSCTP and three types of dNTPs. The natural un-chemically modified device (dsDNA) was prepared using the same protocol but with unmodified dNTPs.

### 4.4. Preparation of DNAs/Liposome-Complexes

Cationic liposomes were prepared using a modified thin-film hydration method as previously reported [16]. Briefly, the lipids; DOPE, cholesterol and DOTAP, were dissolved in chloroform at molar ratios of 3:3:4. After evaporation of chloroform, the thin lipid film was hydrated with 9% sucrose solution at 37 °C to prepare 60 mM lipids suspension. The suspension was sized by repeated extrusion through a polycarbonate membrane filter with pore sizes of 800, 400, 200 and 100 nm. The average size of resulting cationic liposomes was 108.1 ± 3.4 nm, and the zeta-potential was +44.0 ± 0.6 mV (Table 1). The phospholipid concentrations of the resulting liposomes were quantified using colorimetric assay [17]. To form DNA/liposome-complexes, equal volumes of DNAs (pDNA, dsDNA or iRed) and cationic liposomes were mixed at molar ratios of 1:500, 1:800, 1:1000, 1:2000, 1:4000, 1:8000, 1:20,000 or 1:30,000, and vigorously mixed for 5 min at room temperature. Particle sizes and zeta-potentials of prepared liposomes or complexes were determined using a Zetasizer Nano ZS (Malvern Instruments, Worcestershire, UK).

### 4.5. Electrophoresis

Luc pDNA/liposome-complexes and Luc iRed/liposome-complexes, prepared at the above-mentioned molar ratios, were mixed with an equal volume of 20% glycerol and then 14 µL of each mixed sample was applied onto a well of 2% agarose gel containing 0.1 μg/mL ethidium bromide. Electrophoresis was then performed using an electrophoretic devise (PLUS-2, Simabiotech, Chiba, Japan) for 15 min at 100 V. The electrophoresed gel was visualized and digitally photographed using AE-9000N E-Graph (ATTO, Tokyo, Japan).

### 4.6. Cell Culture

A human pleural mesothelioma cell line expressing firefly luciferase (MSTO-211H-Luc) generated via stable transfection with the firefly luciferase gene (pGL3 Basic plasmid, Promega, WI, USA) was kindly provided by Dr. Masashi Kobayashi (Department of Thoracic Surgery, Faculty of Medicine, Kyoto University). The cells were cultured in RPMI-1640 medium (Wako Pure Chemical) supplemented with 10% of fetal bovine serum (FBS), 100 μg/mL streptomycin and 100 units/mL penicillin. The cells were incubated at 37 °C in an atmosphere containing 5% CO_2_.

### 4.7. Preparation of the Orthotopic Mesothelioma Mouse Model

BALB/c nu/nu mice (male, 5 weeks old) were purchased from Japan SLC (Shizuoka, Japan). The experimental animals were allowed free access to water and mouse chow, and were housed under controlled environmental conditions (constant temperature, humidity, and a 12-h dark–light cycle). All animal experiments were approved and conducted in accordance with the guidelines of the Animal and Ethics Review Committee of Tokushima University. For the development of the orthotopic mouse model of mesothelioma, the mice were injected directly into the left pleural cavity with MSTO-211H-Luc cells (1  ×  10^6^ cells/mouse in 100 μL of PBS ).Tumor development in the pleural cavity was monitored using an in vivo imaging system (IVIS, Xenogen, CA, USA). For in vivo imaging, the mice were intraperitoneally injected with 100 μL of 7.5 mg/mL D-luciferin and were subsequently anesthetized using isoflurane inhalation. At 3 min after injection, bioluminescence was recorded using a charge-coupled device camera (1-min exposure). The bioluminescent region of interest (ROI) was calculated and shown as photon counts (photons/s/cm^2^/steradian).

### 4.8. In Vivo Luciferase Reporter Assay

Orthotopic mesothelioma model mice (*n* = 5) were intrapleurally injected with 2 doses of Luc pDNA/liposome-complexes, Luc dsDNA/liposome-complexes, Luc iRed/liposome-complexes or control DNA/liposome-complexes (10 µg DNA/mouse/day) every 3 days starting from Day 7 after tumor cells inoculation. On Day 6, 12, 17 and 22 after the cell inoculation, the luciferase activity of the pleural tumor was observed using IVIS, as described above. The data were represented as relative photon counts compared to that on Day 6. In addition, the inhibition rate of each DNA/liposome-complexes was calculated using the following formula. Body weight changes of the treated mice were monitored every 4 days from Day 6 after the cell inoculation.

Inhibition rate={(1−Relative photon countsSampleRelative photon countsControl)×100}Molar amountsSample

### 4.9. Determination of IL-6 Production

BALB/c nu/nu mice (*n* = 5) were intrapleurally injected with Luc pDNA/liposome-complexes, Luc dsDNA/liposome-complexes or Luc iRed/liposome-complexes (10 µg DNA/mouse). At 4 h post injection, blood was collected from the postcaval vein of the mice. Serum samples were obtained by centrifugation of the blood (3000 rpm, 4 °C, 15 min) following 30-min incubation at room temperature. Pleural wash fluids were prepared by washing the pleural cavity of the treated mice with 0.5 mL PBS (-) containing 0.5% BSA, and the collected fluids were centrifuged at 3000 rpm at 4 °C for 15 min, and then at 30,000 rpm for 10 min to remove cell debris. The level of IL-6 in the serum and the pleural wash fluid was quantified using Mouse IL-6 Quantikine ELISA Kit (R&D Systems, MN, USA) according to the manufacturer’s instruction.

### 4.10. In Vitro Luciferase Reporter Assay

MSTO-211H-Luc cells were seeded in a 12-well plate (5  ×  10^4^ cells/well) and were incubated overnight. The cells were then transfected with Luc pDNA, Luc dsDNA, Luc iRed, or control DNA (0.5 pmol DNA/well) using Lipofectamine^®^ 2000 (Invitrogen, CA, USA) according to the manufacturer’s instruction. Three days post transfection, the cells were washed with PBS (-) and were lysed with passive lysis buffer (Promega, WI, USA). Luciferase activities in the cell lysates were determined using the dual-luciferase reporter assay system (Promega) according to the manufacturer’s instruction. The resultant chemiluminescence was measured using a microplate reader Infinite^®^ 200 PRO (Tecan, Männedorf, Switzerland). The total protein concentration in the cell lysates was measured using the Pierce bicinchoninic acid (BCA) protein assay kit (Thermo Fisher Scientific, MA, USA). The result was reported as the luciferase activity/µg protein.

### 4.11. Quantification of Luciferase shRNA by Real Time RT-PCR

MSTO-211H-Luc cells were seeded in a 12-well plate (5  ×  10^4^ cells/well) and were incubated overnight. The cells were then transfected with Luc pDNA, Luc dsDNA, Luc iRed or control DNA (0.5 pmol DNA/well) using Lipofectamine^®^ 2000 according to the manufacturer’s instructions. At 24, 48 or 72 h post transfection, the cells were washed with PBS and the total RNA was extracted using a Total RNA Extraction Miniprep System (Viogene, CA, USA). The concentrations of the resulting RNA solutions were measured using a NanoDrop™ 8000 (Thermo Fisher Scientific). Reverse transcription reactions were performed using a stem-loop primer for luciferase shRNA and a SuperScript™ III First-Strand Synthesis System (Thermo Fisher Scientific) under the following conditions: 16 °C for 30 min; 60 cycles of synthesis (30 °C for 30 s, 42 °C for 30 s and 50 °C for 1 min) and 85 °C for 5 min. The generated cDNA sample (5 µL) was mixed with a reaction mixture containing 10 µL FastStart Universal Probe Master (Rox; Roche Molecular Systems, CA, USA), 0.045 µL Universal Probe Library (10 µM, Roche Molecular Systems), 0.18 µL forward primer (100 µM), 0.18 µL reverse primer (100 µM) and 4.595 µL water. Real-time PCR was performed using a real-time PCR system (StepOne Plus™, Applied Biosystems, CA, USA) under following conditions: 50 °C for 2 min; 95 °C for 10 min; 40 cycles of amplification (95 °C for 15 sec and 60 °C for 1 min). The data were analyzed using StepOne Software v2.1 (Applied Biosystems). Primer’s sequences are summarized in Supplementary Table S2.

### 4.12. Statistical Analysis

Differences in a group were evaluated by analysis of variance (ANOVA) using BellCurve for Excel software (Social Survey Research Information, Tokyo, Japan). The level of significance was set at * *p* < 0.05, ** *p* < 0.01, *** *p* < 0.001. 

## Figures and Tables

**Table 1:** Physicochemical characteristics of DNAs/liposome-complexes and cationic liposomes. Particle sizes and zeta-potentials were measured using the complexes or liposomes at a concentration of 10 mM lipids.

Complexes	Particle Size (nm)	Zeta Potential (mV)
Luc pDNA/liposome-complexes (molar ratio = 1:30,000)	197.1 ± 0.4	+23.0 ± 2.8
Luc dsDNA/liposome-complexes (molar ratio = 1:2000)	160.9 ± 1.2	+38.0 ± 0.7
Luc iRed/liposome-complexes (molar ratio = 1:2000)	159.1 ± 4.1	+35.5 ± 1.5
Cationic liposomes (DOPE:cholesterol:DOTAP = 3:3:4 molar ratio)	108.1 ± 3.4	+44.0 ± 0.6

**Table 2:** Inhibition rate of DNAs/liposome-complexes calculated from the data in Figure 3b.

Complexes	Inhibition Rate (%/mol)	Significance
Luc pDNA/liposome-complexes	40.4 ± 1.54	*** *p* < 0.001 vs. Luc dsDNA/liposome-complexes *** *p* < 0.001 vs. Luc iRed/liposome-complexes
Luc dsDNA/liposome-complexes	1.0 ± 1.42	
Luc iRed/liposome-complexes	1.65 ± 0.56	

## Supplementary Materials

The following are available online at https://www.mdpi.com/1420-3049/25/7/1725/s1, Supplementary Figure S1: Graphical image of DNAs including Luc pDNA, Luc ds DNA and Luc iRed, Table S1: Sequences of ligated ODNs for construction of pDNAs expressing shRNAs, Table S2: Sequences of primers used for the quantification of transcribed shRNA in MSTO-211H-Luc cells.
