# Supplementary material for: A Unique Gene-Silencing Approach, Using an Intelligent RNA Expression Device (iRed), Results in Minimal Immune Stimulation When Given by Local Intrapleural Injection in Malignant Pleural Mesothelioma
Source: Molecules. 2020 Apr 9;25(7):1725. doi: 10.3390/molecules25071725 (PMC7181240; doi:10.3390/molecules25071725)
Supplement: Supplementary file 1 [file molecules-25-01725-s001.pdf]

Supplementary Figure S1: Graphical image of DNAs including Luc pDNA, Luc ds DNA, and Luc iRed

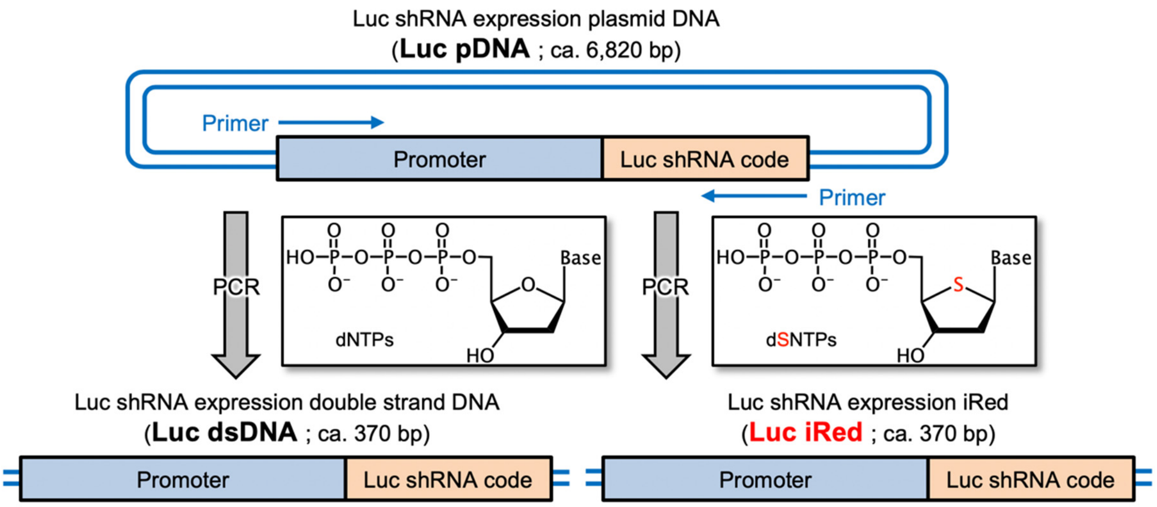

Table S1. Sequences of ligated ODNs for construction of pDNAs expressing shRNAs.

ODNs	Sequence
pU6-pGL2, sense	5’–d(gatccgtgcgttgctagtaccaacttcaagagagttggtactagcaacgcacttttttacgcgtg)–3’
pU6-pGL2, antisense	5’–d(aattcacgcgtaaaaaagtgcgttgctagtaccaactctcttgaagttggtactagcaacgg)–3’
pU6-pGL3, sense	5’–d(gatcccttacgctgagtacttcgatagtgctgctcctggttgtcgaagtactcagcgtaagttttttg)–3’
pU6-pGL3, antisense	5’–d(aattcaaaaaacttacgctgagtacttcgacaaccaggagcagcactatcgaagtactcagcgtaagg)–3’
pU6-NS, sense	5’–d(gatccgtcttaatcgcgtataaggctagtgctcctggttggccttatacgcgattaagattttttg)–3’
pU6-NS, antisense	5’–d(aattcaaaaaatcttaatcgcgtataaggccaaccaggagcactagccttatacgcgattaagacg)–3’

Table S2. Sequences of primers used for the quantification of transcribed shRNA in MSTO-211H cells.

ODNs	Sequence
Stem-loop primer for reverse transcription of luciferase shRNA	5’–d(gttggctctggtgcagggtccgaggtattcgcaccagagccaacaagtgc)–3’
Forward primer for quantitive PCR of luciferase shRNA	5’–d(cgcgcgttggtactagcaac)–3’
Reverse primer for quantitive PCR of luciferase shRNA	5’–d(gtgcagggtccgaggt)–3’
